# Incomplete Refractory Kawasaki Disease in an Infant—A Case Report and a Review of the Literature

**DOI:** 10.3389/fped.2018.00210

**Published:** 2018-07-27

**Authors:** Cristina O. Mărginean, Lorena E. Meliț, Liliana Gozar, Cristian Dan Mărginean, Maria O. Mărginean

**Affiliations:** ^1^Department of Pediatrics, University of Medicine and Pharmacy TîrguMures, Târgu Mures, Romania; ^2^Department of Pediatric Cardiology, University of Medicine and Pharmacy TîrguMures, Târgu Mures, Romania

**Keywords:** Kawasaki disease, coronary aneurysm, methyl-prednisolone, child, refractory form

## Abstract

Kawasaki disease (KD) is a febrile vasculitis, which is commonly defined by fever and at least four specific clinical symptoms. Incomplete KD is defined by suggestive echocardiographic findings with an incomplete clinical picture. Refractory KD is diagnosed in patients resistant to intravenous immunoglobulin (IVIG). We report the case of a 6-month-old male infant admitted to our clinic for persistent fever and onset of a generalized polymorphous rash, accompanied by high fever, rhinorrhea, and cough for the past 7 days. The laboratory tests, on the day of admission, revealed leukocytosis with neutrophilia, anemia, thrombocytosis, hypernatremia, hypoalbuminemia, elevated C-reactive protein (CRP), and erythrocyte sedimentation rate (ESR). Echocardiography showed dilation of the left anterior descending coronary artery (LAD). Based on all these findings, we established the diagnosis of KD, and we initiated IVIG and intravenous pulsed methylprednisolone, with an initial favorable outcome. However, the symptoms reappeared, and we administered a second higher single dose of IVIG, but without any clinical improvement. Moreover, the laboratory parameters and echocardiographic findings worsened. We reinitiated a longer course of intravenous methylprednisolone in a smaller dose, which had a favorable impact on the clinical, laboratory, and echocardiographic parameters. Multiple uncertainties exist related to the management of refractory KD despite the wide spectrum of therapeutic options that have been proposed. Our case demonstrates that in patients refractory to aggressive initial therapy, low or moderate doses of steroid given daily may be helpful.

## Introduction

Kawasaki disease (KD) or mucocutaneous lymph node syndrome was named after Tomisaku Kawasaki, a Japanese pediatrician who described this febrile vasculitis for the first time in 1967 (1). Therefore, common KD involves fever for more than 5 days along with at least four of the following clinical features: erythema of the lips and oral mucosa, bilateral nonexudative conjunctival injection, polymorphous skin rash, changes in the extremities, and unilateral painless cervical lymphadenopathy (2, 3). Incomplete KD refers to patients who do not fulfill all the clinical criteria. In the past, “atypical KD” has also been used to refer to these patients, but this is now felt to be inaccurate. Rather, the term “atypical” should be reserved for patients who express atypical manifestations. In either case, it is not clear that these represent distinct entities within KD, as outcomes have not been shown to be different (2, 4, 5). KD is usually diagnosed in children under the age of 5 years, and the male to female ratio is 1.5:1 (6). The incidence of this condition remains the highest in Japan where it was described for the first time, accounting for ~239 cases in 100,000 children below 5 years of age (7). As for European countries, the data from the United Kingdom reported an annual incidence of 8.4/100,000 in children below 5 years of age whereas, in Denmark and the Netherlands, the incidence is smaller, ~4–5 cases in 100,000 children under the same critical age (8). Although it is well-known that KD is triggered by an infectious agent, its pathogenesis remains unclear. However, recent data strongly support the hypothesis that genetic predisposition is a key factor in favoring susceptibility and severity of this condition (9). Therefore, there is growing evidence that innate immunity has a special role in the determination of acute inflammatory response in KD patients (10). Thus, these patients were found to have high levels of toll-like receptor mRNA and upregulation in interleukin-1 pathway genes (11, 12). Cardiac impairment remains the most severe manifestation of KD. Although the complications can be widely diverse during the acute phase, including valvulitis, myocarditis, pericarditis, or KD shock syndrome, the occurrence of coronary artery aneurysms (CAAs) remains the most important, usually appearing later, in the subacute to convalescent phase (13). CAAs occur in ~25–30% of untreated patients (14).

The clinical course of the disease involves three different stages: acute, subacute, and convalescent phase (13). The diagnosis of KD remains a challenge for pediatricians due to its resemblance with many viral and bacterial illnesses (15). Laboratory tests are usually unspecific for KD, but elevated inflammatory biomarkers, like erythrocyte sedimentation rate (ESR) and C-reactive protein (CRP), as well as leukocytosis with neutrophilia, normocytic normochromic anemia, and thrombocytosis, are associated with the acute phase of this condition (16, 17). Long-term repeated echocardiography is mandatory in KD patients to accurately assess the potential development of cardiac complications. The standard treatment consists of intravenous immunoglobulin (IVIG), recently recommended as a single dose of 2 g/kg of body weight within the first 10 days after the clinical onset of the disease accompanied by initial high doses of oral aspirin of ~80–100 mg/kg/day divided in four doses during the acute phase, which are then lowered to 3–5 mg/kg/day for up to 8 weeks (18). Unfortunately, resistance to IVIG occurs in up to 20% of patients diagnosed with KD (19). However, rescue therapy is not clearly established and differs among hospitals, which report several options, such as intravenous steroids, tumor necrosis factor-alpha inhibitors, plasmapheresis, cyclosporine, cyclophosphamide, and methotrexate (15). These therapeutic options require large-scale prospective studies to determine their safety and efficacy in patients diagnosed with refractory KD. On the other hand, factors that predict refractory KD should be identified to administer an early and appropriate treatment.

The aim of this case report is to underline the challenges associated with the management of refractory KD diagnosed in a young infant.

*Informed consent was obtained from the patient's mother prior to the publication of this case report*.

## Case report

### Presenting concerns

We report the case of a 6-month-old male infant admitted to our clinic for persistent fever and a generalized polymorphous rash. The onset of the disease, with fever, rhinorrhea, and cough was ~7 days before the admission. Therefore, he was admitted to a regional hospital where he benefited from antibiotics and antipyretics, but there was no improvement. He also presented with a generalized polymorphous rash and bilateral nonexudative conjunctival injection and was transferred to our clinic with suspected KD.

### Clinical findings

**Figure 1:**
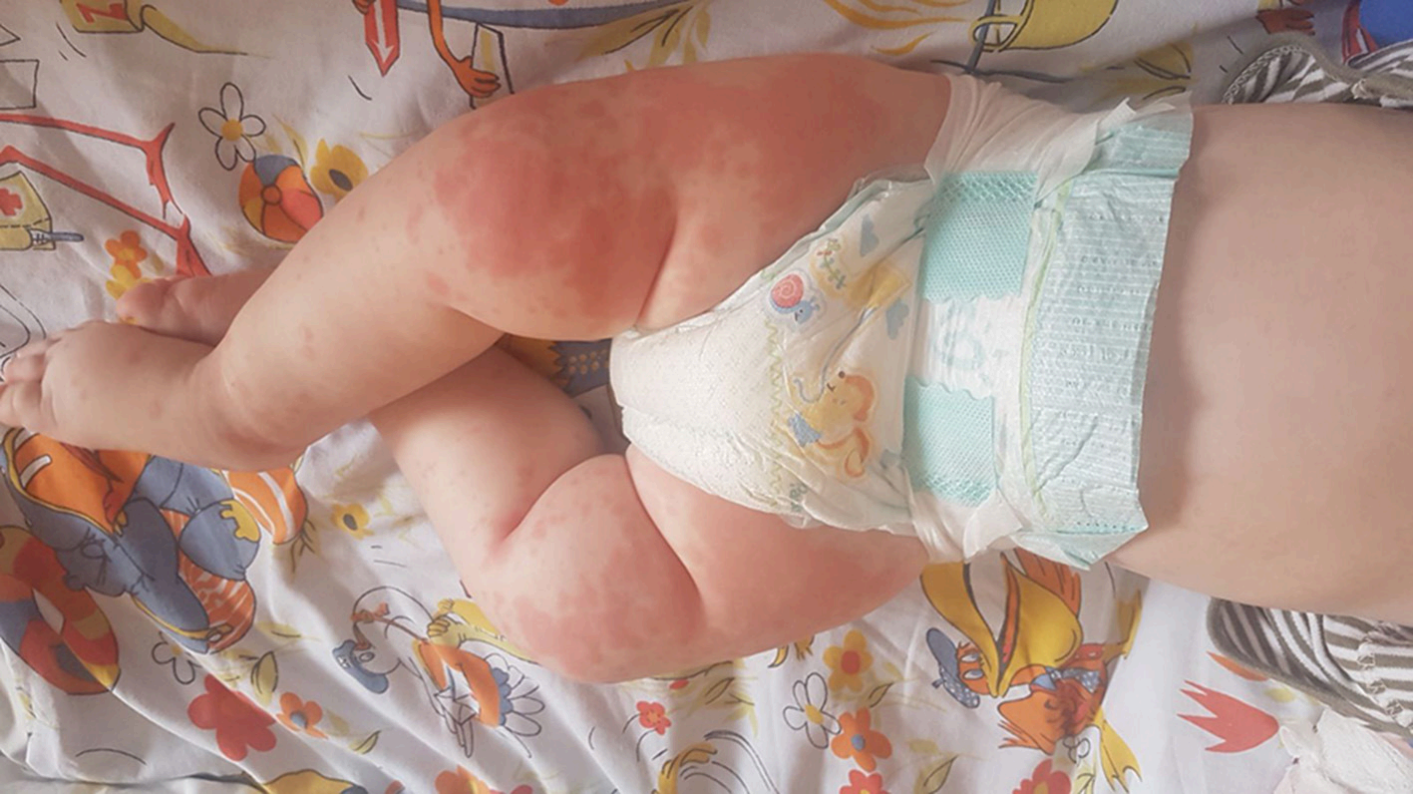
Polymorphous rash on the infant's inferior limbs.

The clinical exam revealed the following pathological elements at the time of admission: influenced general status, pallor, a polymorphous rash on the limbs and face (Figure 1), bilateral conjunctival hyperemia, painless right cervical lymphadenopathy, and a productive cough.

### Diagnostic focus and assessment

**Figure 2:**
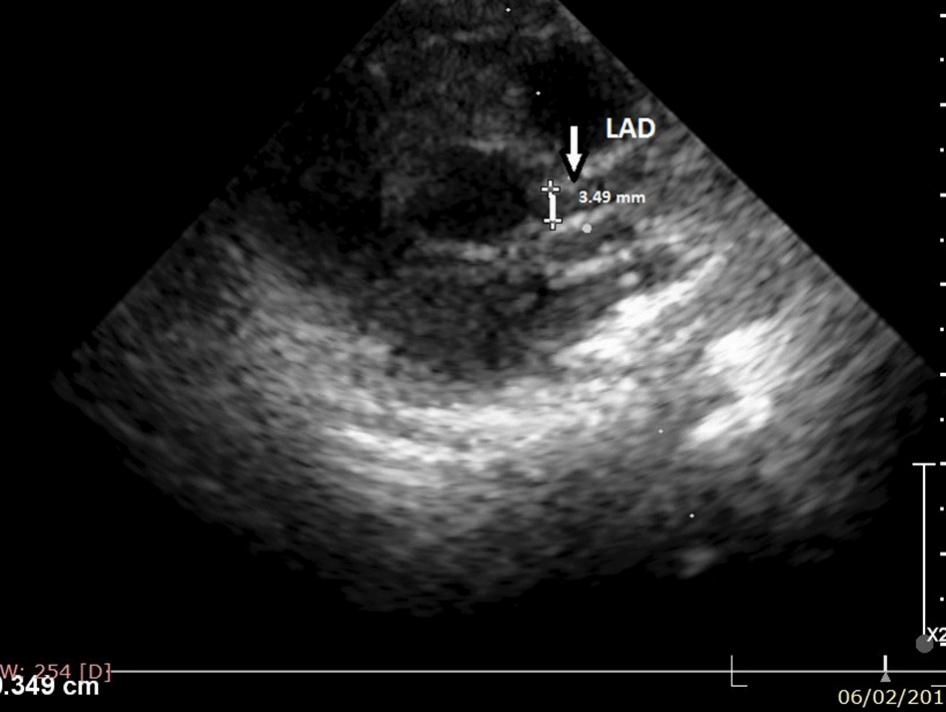
Cross-sectional view at the level of the great arteries: left anterior descending (LAD) artery (at 3 o'clock).

The laboratory tests on the day of admission revealed leukocytosis (34,590/μl) with neutrophilia (28,000/μl), anemia (Hb: 7.5 g/dl, Htc: 23.5%, MEV: 73 fl, MEH: 23.3 pg), thrombocytosis (648,000/μl), hypernatremia (154.1 mmol/l), hypoalbuminemia (2.48 g/dl), elevated CRP (311.33 mg/l), and ESR (65 mm/h). The urinary exam and blood culture were negative. The initial echocardiography showed good ventricular contractility, diastolic dysfunction, mild mitral regurgitation and moderate dilatation of LAD (the internal diameter was 3.49 mm and Z score + 7.62). (Figure 2). The abdominal ultrasound revealed a right renal cyst without pathological elements. Based on all these findings, we established the diagnosis of KD.

### Therapeutic focus and assessment

Due to the echocardiographic findings, we initiated IVIG in a dose of 400 mg/kg/day for 5 days accompanied by intravenous pulsed methylprednisolone at 30 mg/kg/day for 3 days and high doses of aspirin at 100 mg/kg/day. We also administered substitution with erythrocyte mass and human albumin.

### Follow-up and outcome

**Figure 3:**
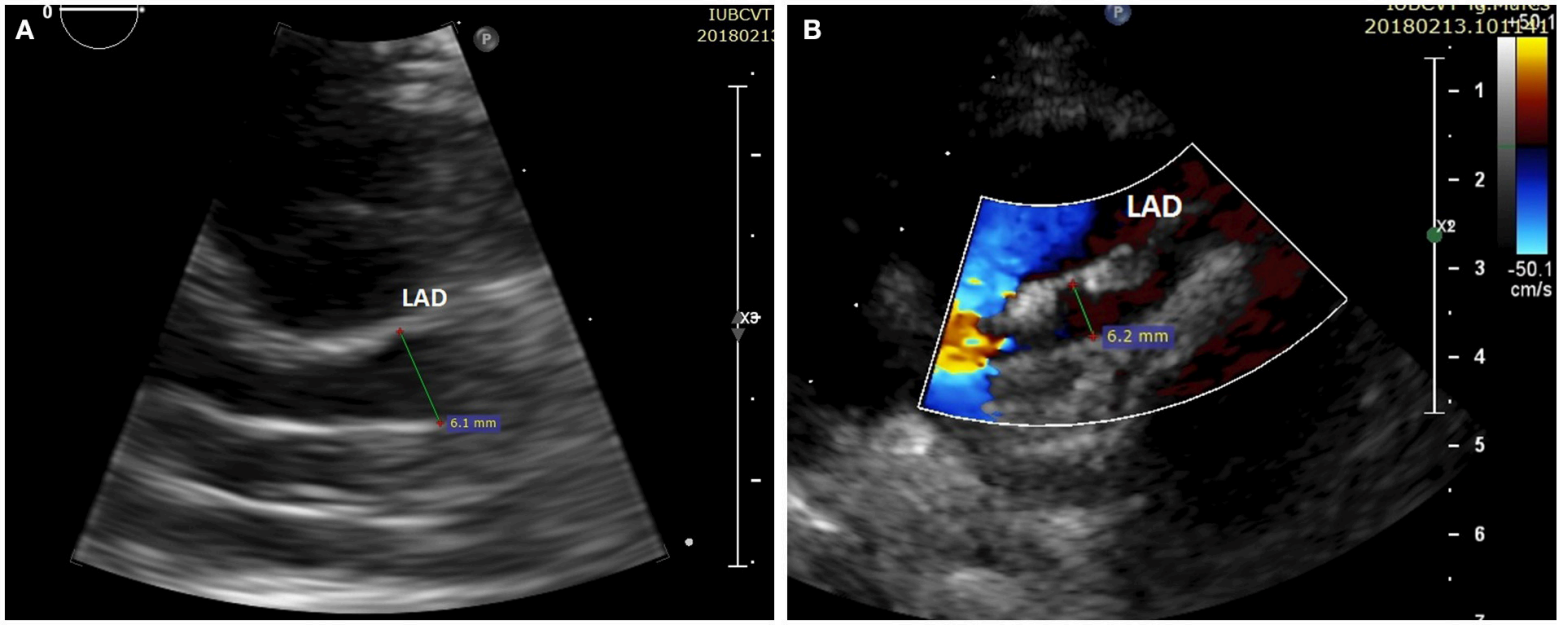
**(A)** Cross-sectional view at the level of the great arteries: left anterior descending (LAD) with an aneurysm (diameter > 6 mm, Z score + 16.45). **(B)** The same image as **3A** with color Doppler.

The clinical symptoms and laboratory parameters improved within the first days after the initiation of the above-mentioned treatment, but unfortunately, after ~1 week from the cessation of IVIG treatment, the fever and the bilateral conjunctival injection reappeared. The echocardiographic re-evaluation showed an aneurysm of the LAD with internal diameter 6.2 mm, Z score + 16.45 (Figures 3A, B) with a hyperechoic image inside raising the suspicion of a thrombus or a thickening of the coronary lumen. The cardiologist recommended the initiation of low-molecular heparin in the treatment, and the lowering of the aspirin dose at 5 mg/kg/day in a single dose. We performed an angio-CT scan that confirmed a potential thrombus. We also repeated the laboratory parameters and found increased levels of CRP. Therefore, we decided to administer another IVIG in a single higher dose of 2 g/kg, but the inflammatory biomarker remained elevated. Based on all these findings we decided to re-initiate intravenous methylprednisolone, but in a lower dose, of 1.5 mg/kg/day twice a day for ~1 week. The ESR values started to decrease progressively, and therefore we switched to oral methylprednisolone tapering the dose gradually for ~3 weeks. The echocardiographic re-evaluation did not reveal any improvement, and for this reason, the cardiologist recommended the continuation of the low-molecular heparin for ~6 weeks and aspirin for 3 months. After ~2 months, the infant's status generally improved, but the echocardiography underlined a persistent dilation of the left coronary artery with an aneurysmal portion of ~6 mm and a tendency of stenosis below this portion.

## Discussion

KD remains a challenging entity in pediatrics regarding both the diagnosis and management, especially in the cases of refractory KD. Recent studies focus on identifying the etiology of KD and the risk factors involved in its development. Therefore, a recent study suggested that innate immune cells have the ability to develop a memory with significant implications in the host's response to pathogens and autoimmune disorders (20). Even though refractory KD is common and is usually associated with CAAs, there are no clear factors available to predict its development. Nevertheless, different scoring systems have been defined to predict non-responsiveness to initial IVIG therapy (21–23). Additionally, a high serum ferritin level was found to be associated with refractory KD, and it could be useful in patients in whom sensitivity of the scoring systems is low (24). On the other hand, several other risk factors were identified, which favor the development of CAAs, including male gender, prolonged fever, delay in diagnosis or treatment, and extremes of age (18). Our patient fulfilled two of these conditions, namely male gender and extremes of age, being a 6-month-old infant. Similarly, the administration of IVIG, aspirin, and warfarin during the first 10 days after the onset was found to lower the risk of CAA development (25). Despite receiving both IVIG and high doses of aspirin within the first 10 days after onset, our patient developed an aneurysm of the left coronary artery. Although we did not initiate warfarin, we did administer low-molecular heparin, though not within the first 10 days after the onset.

The early diagnosis of KD is hindered by the important similitudes between its clinical appearance and that of other infectious diseases. Therefore, in these cases, echocardiography can be helpful. A normal echocardiography within the first 7 days of fever cannot rule out KD, but positive findings can confirm the diagnosis (26). Incomplete KD usually appears in young infants, and it must never be mistaken as a mild form of KD (26). Our case also presented an incomplete form of KD because, in addition to fever, he had only three clinical features: polymorphous rash, bilateral nonexudative conjunctival injection and right painless cervical lymphadenopathy. Also, similar to the data mentioned above, he was a young infant. Despite this incomplete presentation, the diagnosis in our case was not delayed more than 10 days, the treatment being initiated after ~7 days with both IVIG and intravenous methylprednisolone due to the positive echocardiographic findings. Similarly, a very recent case report described an incomplete KD in a 4-year-old male, with known hypogammaglobulinemia, who presented only fever associated with conjunctival hyperemia and membranous desquamation of fingertips of hands and feet (27). The authors hypothesized that hypogammaglobulinemia could be a leading cause of incomplete KD (27). In contrast, our case did not present with any disorders of the immune system. Laboratory tests are unspecific but patients with KD can present the following pathological elements: high ESR and CRP, leukocytosis with neutrophilia, normocytic normochromic anemia, and thrombocytosis (16, 17). Our patient presented elevated inflammatory biomarkers, leukocytosis with neutrophilia, thrombocytosis, anemia, and he also had hypoalbuminemia. Hyponatremia was also identified as a negative predictive marker for adverse coronary outcomes (16). Although in our case we found early impairment of the left coronary artery, the patient presented with hyper-, and not, hyponatremia.

The management of refractory KD patients has not yet been clearly established. Multiple therapeutic options have been postulated, but no general consensus has yet been defined. Nevertheless, the guidelines recommend the use of a second dose of IVIG or steroids in refractory KD (28). In our case, we preferred to administer the immunoglobulin divided into 5 doses for consecutive 5 days and not in a single dose because we also associated intravenous methylprednisolone pulsed therapy even since the first day. Therefore, we considered that we were aggressive enough by administering both IVIG and methylprednisolone pulsed therapy. The use of steroids in refractory KD is based on their ability to downregulate inflammatory mediators, to hinder leukocyte migration and to decrease capillary permeability (29). It was also proved that in case of patients that do not respond to IVIG, the incidence of CAAs was lower for those who received both IVIG and prednisolone than for the groups that were administered either prednisolone or IVIG alone (30). These results were also supported by the findings of Inoue et al. (31). Moreover, another important study in the literature, “Randomized controlled trial to Assess Immunoglobulin plus Steroid Efficacy for Kawasaki disease” (RAISE), performed by Kobayashi et al. assessed the efficacy of the combination of IVIG, aspirin, and prednisolone for 5 days intravenously followed by tapered oral doses for up to 3 weeks (32). The authors found a significantly lower incidence of CAAs in case of the group who received IVIG plus prednisolone than in the IVIG alone group, not just during the study period but also after four weeks (32). We also administered intravenous pulsed methylprednisolone therapy with the initial dose of IVIG, but the symptoms persisted. Thus, we attempted a second dose of 2 g/kg IVIG, but the levels of the inflammatory biomarkers continued to increase. Eventually, we reinitiated intravenous methylprednisolone in a smaller dose of 1.5 mg/kg twice a day for ~7 days achieving a gradually favorable outcome, followed by tapered oral doses for three weeks. However, it is well-known that glucocorticoid therapy may present multiple side effects, such as gastrointestinal bleeding, infection, secondary adrenocortical insufficiency, hypercoagulability, which must be prevented if possible or closely monitored (33). Overall, steroids could be used in case of acute KD patients that did not respond to IVIG or even included with IVIG in those who are predicted non-responders (33). The prognosis for KD patients is burdened by its long-term cardiovascular outcome, and it seems that CAAs can occur in untreated patients, as well as in ~5% of those who are appropriately managed. Additionally, CAAs greater than 6 mm are strongly related to myocardial ischemia (34). Similarly, our patient developed a left coronary aneurysm of more than 6 mm independent of the adequately aggressive treatment, which was administered as early as possible.

## Conclusions

Incomplete KD is a challenging diagnosis that usually appears in young infants, being linked to CAA development. Refractory KD presents a wide range of difficulties regarding the appropriate management of these patients. The rescue therapy has not yet been clearly established, but our case demonstrates that in patients refractory to aggressive initial therapy, low or moderate doses of steroid given daily may be helpful.

## Author contributions

CM, LM, and MM conceptualized and designed the study, drafted the initial manuscript, and reviewed and revised the manuscript. LG and CDM designed the data collection instruments, collected data, carried out the initial analyses, and reviewed and revised the manuscript. All authors approved the final manuscript as submitted and agree to be accountable for all aspects of the work.

### Conflict of interest statement

The authors declare that the research was conducted in the absence of any commercial or financial relationships that could be construed as a potential conflict of interest.

## Abbreviations

CAAs: coronary artery aneurysms
CRP: C-reactive protein
ESR: erythrocyte sedimentation rate
Hb: hemoglobin
Htc: hematocrit
IVIG: intravenous immunoglobulin
KD: Kawasaki disease
LAD: left anterior descending coronary artery
MEV: medium erythrocyte volume
MEH: medium erythrocyte hemoglobin.

## References

[1] Kontopoulou T Kontopoulos DG Vaidakis E Mousoulis GP. Adult Kawasaki disease in a European patient: a case report and review of the literature. J Med Case Rep. (2015) 9:75. 10.1186/s13256-015-0516-9 25890055 PMC4403952

[2] Newburger JW Takahashi M Gerber MA Gewitz MH Tani LY Burns JC. Diagnosis, treatment, and long-term management of Kawasaki disease: a statement for health professionals from the committee on rheumatic fever, endocarditis and kawasaki disease, council on cardiovascular disease in the young, American Heart Association. Circulation (2004) 110:2747–71. 10.1161/01.CIR.0000145143.19711.78 15505111

[3] Freeman AF Shulman ST. Kawasaki disease: summary of the American Heart Association guidelines. Am Fam Physician. (2006) 74:1141–8. 17039750

[4] Sánchez-Manubens J Bou R Anton J. Diagnosis and classification of Kawasaki disease. J Autoimmun. (2014) 48-49:113–17. 10.1016/j.jaut.2014.01.010 24485156

[5] McCrindle BW Rowley AH Newburger JW Burns JC Bolger AF Gewitz M. Diagnosis, treatment, and long-term management of Kawasaki disease: a scientific Statement for Health Professionals from the American Heart Association. Circulation (2017) 135:e927–99. 10.1161/CIR.0000000000000484 28356445

[6] Singh S Vignesh P Burgner D. The epidemiology of Kawasaki disease: a global update. Arch Dis Child. (2015) 100:1084–8. 10.1136/archdischild-2014-307536 26111818

[7] Greco A De Virgilio A Rizzo MI Tombolini M Gallo A Fusconi M. Kawasaki disease: an evolving paradigm. Autoimmun Rev. (2015) 14:703–9. 10.1016/j.autrev.2015.04.002 25882057

[8] Luca NJC Yeung RSM. Epidemiology and management of Kawasaki disease. Drugs (2012) 72:1029–38. 10.2165/11631440-000000000-00000 22621692

[9] Newburger JW Takahashi M Burns JC. Kawasaki Disease. J Am Coll Cardiol. (2016) 67:1738–49. 10.1016/j.jacc.2015.12.073 27056781

[10] Hara T Nakashima Y Sakai Y Nishio H Motomura Y Yamasaki S. Kawasaki disease: a matter of innate immunity. Clin Exp Immunol. (2016) 186:134–43. 10.1111/cei.12832 27342882 PMC5054572

[11] Huang YH Li SC Huang LH Chen PC Lin YY Lin CC. Identifying genetic hypomethylation and upregulation of Toll-like receptors in Kawasaki disease. Oncotarget. (2017) 8:11249–58. 10.18632/oncotarget.14497 28061462 PMC5355262

[12] Hoang LT Shimizu C Ling L Naim AN Khor CC Tremoulet AH. Global gene expression profiling identifies new therapeutic targets in acute Kawasaki disease. Genome Med. (2014) 6:541. 10.1186/s13073-014-0102-6 25614765 PMC4279699

[13] Weiss PF. Pediatric vasculitis. Pediatr Clin North Am. (2012) 59:407–23. 10.1016/j.pcl.2012.03.013 22560577 PMC3348547

[14] Uehara R Belay ED. Epidemiology of Kawasaki disease in Asia, Europe, and the United States. J Epidemiol. (2012) 22:79–85. 10.2188/jea.JE20110131 22307434 PMC3798585

[15] Agarwal S Agrawal DK. Kawasaki disease: etiopathogenesis and novel treatment strategies. Expert Rev Clin Immunol. (2017) 13:247–58. 10.1080/1744666X.2017.1232165 27590181 PMC5542821

[16] Patel RM Shulman ST. Kawasaki disease: a comprehensive review of treatment options. J Clin Pharm Ther. (2015) 40:620–5. 10.1111/jcpt.12334 26547265

[17] Scuccimarri R. Kawasaki disease. Pediatr Clin North Am. (2012) 59:425–45. 10.1016/j.pcl.2012.03.009 22560578

[18] Bayers S Shulman ST Paller AS. Kawasaki disease: part II. Complications and treatment. J Am Acad Dermatol. (2013) 69:513.e1-8; quiz 521–2. 10.1016/j.jaad.2013.06.040 24034380

[19] Nakagama Y Inuzuka R Hayashi T Eryu Y Shindo T Isoda T. Fever pattern and C-reactive protein predict response to rescue therapy in Kawasaki disease. Pediatr Int (2016) 58:180–4. 10.1111/ped.12762 26222760

[20] Chen KYH Messina N Germano S Bonnici R Freyne B Cheung M. Innate immune responses following Kawasaki disease and toxic shock syndrome. PloS ONE. (2018) 13:e0191830. 10.1371/journal.pone.0191830 29447181 PMC5813928

[21] Kobayashi T Inoue Y Takeuchi K Okada Y Tamura K Tomomasa T. Prediction of intravenous immunoglobulin unresponsiveness in patients with Kawasaki disease. Circulation (2006) 113:2606–12. 10.1161/CIRCULATIONAHA.105.592865 16735679

[22] Egami K Muta H Ishii M Suda K Sugahara Y Iemura M. Prediction of resistance to intravenous immunoglobulin treatment in patients with Kawasaki disease. J Pediatr. (2006) 149:237–40. 10.1016/j.jpeds.2006.03.050 16887442

[23] Sano T Kurotobi S Matsuzaki K Miki K Matsuzaki K Matsuoka T. Prediction of non-responsiveness to standard high-dose gamma-globulin therapy in patients with acute Kawasaki disease before starting initial treatment. Eur J Pediatr. (2007) 166:131–7. 10.1007/s00431-006-0223-z 16896641

[24] Yamamoto N Sato K Hoshina T Kojiro M Kusuhara K. Utility of ferritin as a predictor of the patients with Kawasaki disease refractory to intravenous immunoglobulin therapy. Mod Rheumatol. (2015) 25:898–902. 10.3109/14397595.2015.1038430 25849851

[25] Soriano-Ramos M Martínez-Del Val E Negreira Cepeda S González-Tomé MI Cedena Romero P Fernández-Cooke E. Risk of coronary artery involvement in Kawasaki disease. Arch Argent Pediatr. (2016) 114:107–13. 10.5546/aap.2016.eng.107 27079387

[26] Jiao F Jindal AK Pandiarajan V Khubchandani R Kamath N Sabui T. The emergence of Kawasaki disease in India and China. Glob Cardiol Sci Pract. (2017) 2017:e201721. 10.21542/gcsp.2017.21 29564342 PMC5856971

[27] Sanlidag B Balkan C Bahçeciler N. A case report: incomplete Kawasaki disease in a hypogammaglobulinémie child. Arch Argent Pediatr. (2018) 116:e322–4. 10.5546/aap.2018.eng.e322 29557626

[28] Portman MA Olson A Soriano B Dahdah N Williams R Kirkpatrick E. Etanercept as adjunctive treatment for acute Kawasaki disease: study design and rationale. Am Heart J. (2011) 161:494–9. 10.1016/j.ahj.2010.12.003 21392603

[29] Saneeymehri S Baker K So TY. Overview of pharmacological treatment options for pediatric patients with refractory kawasaki disease. J Pediatr Pharmacol Ther. (2015) 20:163–77. 10.5863/1551-6776-20.3.163 26170768 PMC4471710

[30] Kobayashi T Kobayashi T Morikawa A Ikeda K Seki M Shimoyama S. Efficacy of intravenous immunoglobulin combined with prednisolone following resistance to initial intravenous immunoglobulin treatment of acute Kawasaki disease. J Pediatr. (2013) 163:521–6. 10.1016/j.jpeds.2013.01.022 23485027

[31] Inoue Y Okada Y Shinohara M Kobayashi T Kobayashi T Tomomasa T. A multicenter prospective randomized trial of corticosteroids in primary therapy for Kawasaki disease: clinical course and coronary artery outcome. J Pediatr. (2006) 149:336–41. 10.1016/j.jpeds.2006.05.025 16939743

[32] Kobayashi T Saji T Otani T Takeuchi K Nakamura T Arakawa H. Efficacy of immunoglobulin plus prednisolone for prevention of coronary artery abnormalities in severe Kawasaki disease (RAISE study): a randomised, open-label, blinded-endpoints trial. Lancet Lond Engl. (2012) 379:1613–20. 10.1016/S0140-673661930-2 22405251

[33] Miura M. Role of glucocorticoids in Kawasaki disease. Int J Rheum Dis. (2018) 21:70–5. 10.1111/1756-185X.13209 29105310

[34] Lee H Shin J Eun L. Myocardial assessment in school-aged children with past kawasaki disease. J Korean Med Sci. (2017) 32:1835–9. 10.3346/jkms.2017.32.11.1835 28960037 PMC5639065

